# Social media influence on COVID-19 vaccine perceptions among University students: a Malawi case study

**DOI:** 10.1186/s12889-024-18764-8

**Published:** 2024-05-14

**Authors:** Mervis Folotiya, Chimwemwe Ngoma

**Affiliations:** 1 Malawi University of Business and Applied Sciences, Blantyre, Malawi; 2 Department of Research and Innovation, ThinkSmart Consulting, Lilongwe, Malawi

**Keywords:** COVID-19, Vaccination, Social media, University students, Malawi, Vaccine perceptions, Communication strategies

## Abstract

**Introduction:**

The global fight against the COVID-19 pandemic relies significantly on vaccination. The collective international effort has been massive, but the pace of vaccination finds hindrance due to supply and vaccine hesitancy factors. Understanding public perceptions, especially through the lens of social media, is important. This study investigates the influence of social media on COVID-19 vaccine perceptions among university students in Malawi.

**Methods:**

The study utilized a quantitative methodology and employed a cross-sectional study design to explore the relationship between social media dynamics and COVID-19 vaccine perceptions among 382 randomly sampled students at MUBAS. Data, collected by use of a Likert-scale questionnaire, was analyzed using IBM SPSS 20 for descriptive statistics and Pearson correlation tests.

**Results:**

The findings reveal crucial correlations. Specifically, trust in online vaccine information shows a positive correlation (*r* = 0.296, *p* < 0.01) with active engagement in social media discussions. Conversely, a negative correlation surfaces concerning individuals’ reactions to vaccine availability in Malawi (*r* = -0.026, *p* > 0.05). The demographic overview highlights the prevalence of the 16 to 30 age group, representing 92.9% of respondents.

**Conclusions:**

The identified correlations emphasize the need for careful communication strategies tailored to combat misinformation and enhance vaccine acceptance among the younger demographic in Malawi. The positive correlation between trust in online vaccine information and social media engagement underscores digital platforms’ potential for disseminating accurate information. Conversely, the negative correlation with vaccine availability reactions suggest the presence of complex factors shaping public perceptions.

## Introduction

Vaccination has become an important weapon in the global fight against the Coronavirus Disease 2019 (COVID-19), helping to slow down the virus’s spread [1]. Global efforts to implement COVID-19 vaccination campaigns have been unprecedented in scale and scope [2], with governments, pharmaceutical firms, and organizations collaborating to develop, manufacture, and distribute vaccines at a faster pace [3]. However, the difficulties have been complex, involving issues like vaccine hesitancy and unequal vaccine supply and distribution [4].

The pandemic has had an impact on Malawi’s healthcare delivery and public health interventions, primarily due to the country’s limited healthcare infrastructure, socio-economic disparities, and cultural beliefs that influence perceptions of healthcare practices [5, 6]. This context has also contributed to vaccine hesitancy, resulting in a lower uptake of COVID-19 vaccines in the country. As of May 2023, the uptake rate stands at 40%, which is lower than the country’s 60% target [7, 8].

A recent scoping review on COVID-19 vaccination hesitancy among Malawians reveals that COVID-19 vaccine reluctance is primarily the result of misinformation, with vaccines perceived as harmful or dangerous. Myths such as infertility, severe disability, or even death have contributed to vaccine hesitancy [9]. The review also reveals that some people refuse vaccinations because of their religious convictions and beliefs [9]. The challenges posed by vaccine hesitancy in Malawi highlight the need for targeted communication strategies and public health initiatives that consider the country’s unique socio-cultural context, aimed at achieving widespread vaccination coverage and understanding public opinions about COVID-19 vaccinations [8, 10, 11]. These strategies must address factors such as misinformation, lack of trust in healthcare institutions, fear of side effects, and cultural beliefs surrounding vaccination, which contribute to reluctance to accept COVID-19 vaccines.

Vaccine perception plays an important role in determining the success of vaccination efforts, and these perceptions are shaped by exposure to (mis)information amplified by the media, the community, and the health system. Notably, social networks may either positively or negatively impact vaccination uptake, depending on their views on vaccines [12]. Given these challenges, the success of vaccination campaigns relies not only on the development and distribution of vaccines but also on how these interventions are perceived by the public. Public attitudes and beliefs surrounding vaccine safety, efficacy, and necessity significantly impact vaccine uptake [11].

Social media has emerged a powerful tool in the current information-dissemination landscape for influencing public opinion. Its role in health communication has expanded significantly, providing a dynamic platform for sharing information, influencing attitudes, and shaping behavior [13]. During the COVID-19 pandemic, social media platforms played an important role in amplifying public health messages [14]. However, social media’s very nature, which is marked by a rapid flow of information and a variety of sources, also poses numerous challenges. The spread of false and scientifically inaccurate information are some of the issues that health communication must deal with in the digital age [15].

Within the unique university settings, the dynamics of vaccine perceptions take on a distinctive dimension. The convergence of diverse backgrounds, cultures, and perspectives among university students creates a rich tapestry of attitudes towards health-related issues. Understanding the specific distinctions within this demographic is crucial for tailoring effective public health interventions. Factors such as lack of access, affordability, health disparities, educational background, peer pressure, political views, and lack of trust in institutions may be influenced in unique ways [16].

Despite the growing body of literature exploring the influence of social media on vaccine perceptions, a research gap exists concerning its specific impact on COVID-19 vaccine perceptions among university students in Malawi. While other studies have examined aspects of social media influence on COVID-19 perceptions [17, 18, 19, 20], such as the role of trust in online vaccine information, and engagement in social media discussions, there remains a need for more research within this specific demographic. Therefore, this study seeks to contribute in bridging this gap by providing valuable insights that not only enhance the academic understanding of the subject but also provide practical implications for public health communication strategies tailored to the Malawian university setting.

## Methods

The study employed a quantitative methodology, and utilized a cross-sectional study design to investigate the relationship between social media dynamics and COVID-19 vaccine perceptions among university students at Malawi University of Business and Applied Sciences (MUBAS), which had a student population of 7,619 during the 2022/2023 academic year.

### Sampling

To ensure unbiased participant selection and equitable representation, a simple random sampling technique was employed. Using the population of students at MUBAS, a sampling frame was created in Excel, including student IDs as the unique identifier number. Subsequently, a random number generator was integrated in Excel to randomly select participants in the study to ensure that each participant had an equal chance of being selected. Using the Taro Yamane method, a sample size of 380 respondents was determined from the total student population of 7619, at precision level of 5%. However, for practical considerations, the sample size was adjusted upwards to 388. Potential participants were approached through an invitation process, and were informed about the purpose of the study. A total of 382 complete questionnaires were collected.

### Data collection

A self-administered questionnaire, organized into three sections (demographics, access to COVID-19 vaccine information on social media, and awareness of COVID-19 vaccine information), was used for data collection. Respondents’ attitudes and perspectives were recorded using the Likert scale. The data collection process involved a comprehensive exploration of variables, covering aspects such as social media usage, access to COVID-19 vaccine information, engagement levels, trust, and demographic details. The survey yielded a response rate of 99.5%, indicating robust participant engagement and contributing to the reliability of the gathered data. Ethical considerations, including confidentiality, consent, and voluntary participation, were observed to safeguard the privacy of the participants. Additionally, the study was evaluated and approved by the MUBAS Postgraduate Research Evaluation Committee, study number MMS/20/PG/004.

### Data analysis

Rigorous editing procedures were applied to ensure data completeness and consistency. IBM SPSS 20 software was used for data coding, cleaning, and analysis. Descriptive statistics and Pearson correlation tests were also performed to derive insights from the dataset. In-depth analyses involved the interpretation of demographic data, an assessment of social media dynamics, and exploration of relationships through Pearson correlation. Results were interpreted based on statistical significance, effect size, and alignment with existing research, contributing to a better understanding of the interaction between social media, COVID-19 vaccine misinformation, and hesitancy among university students.

## Results

The study established that all participants in the study utilized social media and internet for various purposes. A noteworthy positive correlation (*r* = 0.296, *p* < 0.01) emerged, indicating a strong association between trust in online vaccine information and active engagement in social media discussions. Conversely, a significant negative correlation (*r* = -0.610, *p* < 0.01) was identified, shedding light on the relationship between individuals’ reactions to vaccine availability in Malawi and their trust in online information.

### Demographic overview

This section provides an overview of the respondents’ demographics using descriptive statistics. Four key characteristic namely age, education year, gender, and nationality, were analyzed to unveil insights into the composition of the study participants.

The study participants exclusively comprised Malawian students, aligning with the university’s predominantly undergraduate student population, which constitutes a significant percentage of the study sample. International students registered at the university are mostly postgraduate students and represented by less than 5% of the study population. Respondents covered various age groups, with the majority falling within the 21 to 30 years range (*n* = 230). This age bracket represents 60.2% of the total respondents. 26.7% of the respondents were in their fourth year, 25.4% in the first year, and 21.7% and 21.2% in the second and third years, respectively. Postgraduate students represented the smallest group at 5%. Regarding gender, 50.3% of respondents were female, while 49.7% were male.

### Correlation matrix of key variables

**Note**

- Correlation is deemed significant at the 0.01 level (two-tailed).
- *N* = 382 for all correlations.

Table 1 presents an analysis of key variables related to COVID-19 vaccine perceptions among university students. The Pearson correlation coefficient shows the relationship between trust in online COVID-19 vaccine information, COVID-19 vaccine effectiveness, participation in COVID-19 discussions on social media, and response to the COVID-19 vaccine.

**Table 1:** Pearson Correlation matrix of key variables related to covid-19 vaccine perceptions

		Trust in online COVID 19 vaccine information	COVID 19 Vaccinated	Engagement in COVID-19 discussion on social media	people’s reaction on COVID-19 vaccine availability in Malawi
Trust in online COVID 19 vaccine information	Pearson Correlation	1	-0.095	0.296^**^	-0.026
	Sig. (2-tailed)		0.064	0	0.614
	N	382	382	382	382
COVID 19 Vaccinated	Pearson Correlation	-0.095	1	-0.087	− 0.610^**^
	Sig. (2-tailed)	0.064		0.089	0
	N	382	382	382	382
Engagement in COVID-19 discussion on social media	Pearson Correlation	0.296^**^	-0.087	1	-0.074
	Sig. (2-tailed)	0	0.089		0.15
	N	382	382	382	382
people’s reaction on COVID-19 vaccine availability in Malawi	Pearson Correlation	-0.026	− 0.610^**^	-0.074	1
	Sig. (2-tailed)	0.614	0	0.15	
	N	382	382	382	382

A noteworthy positive correlation (*r* = 0.296, *p* < 0.01) between trust in online vaccine information and participation in social media discussions is observed, indicating that people who trust online vaccine information are more likely to participate in digital health discussions. On the other hand, a significant correlation (*r* = -0.610, *p* < 0.01) between trust in internet-based vaccine information and individuals’ attitudes toward vaccination, highlighting the influence of trust on vaccination perceptions. Additionally, a modest negative correlation (*r* = -0.087, *p* > 0.05) between the COVID-19 vaccine and participating in Covid-19 discussions on social media is noted. Although not statistically significant, this relationship suggests a possible trend by which individuals protected from COVID-19 may exhibit lower levels of social media engagement.

## Discussion

The demographic overview in Table 2 reveals that a majority of respondents (*n* = 230), fall within the 21 to 30 age range. Notably, the 16 to 30 age range constitutes an accumulative 92.9% of the total respondents. This aligns with predominant internet usage patterns globally [21], and emphasizes the importance of understanding the perspectives of this demographic in shaping COVID-19 vaccine perceptions. Customizing information dissemination to this group’s preferences and habits becomes important, since they make up a significant portion of social media and internet users.

**Table 2:** Demographic characteristics of participants

Characteristics	Category	Frequency (*n*)	Percentage (%)
Age range	16 to 20	125	32.7
	21 to 30	230	60.2
	31 to 40	17	4.5
	41 above	10	2.6
Year of study	First year	97	25.4
	Second year	83	21.7
	Third year	81	21.2
	Fourth year	102	26.7
	Postgraduate	19	5
Gender	Male	190	49.7
	Female	192	50.3

The gender distribution in Table 2, shows a near-balanced representation, with 50.3% female and 49.7% male respondents. Previous research on the connection between gender and social media use reveal broader trends indicating the popularity of social media among females than males [22–24]. The study benefits from the diverse perspectives contributed by both genders in social media discussions, which is highlighted by this balance.

The study examined the relationships among the variables influencing the perceptions of COVID-19 vaccines. The analysis in Table 1 reveals a positive correlation between trust in online vaccine information and active engagement in COVID-19 discussions on social media (*r* = 0.296, *p* < 0.01). This aligns with existing literature, which suggests that people who place trust in online health information are more likely to actively participate in digital health dialogues [25]. Furthermore, the relationship between trust and engagement extends beyond mere participation, it influences the dissemination of accurate information and the formation of informed opinions within online communities [26].

One effective way to promote trust in online vaccine information is through transparent communication practices, including providing information from reputable sources, ensuring data accuracy and reliability, engaging with credible health experts, and conducting educational campaigns on health literacy and critical thinking [27].

Moreover, the positive correlation emphasizes the potential of digital platforms, particularly social media, in promoting health literacy and influencing public health behaviors [28]. As individuals trust the information they encounter online, they are more likely to share it with their social networks, leading to broader awareness and understanding of vaccination-related issues [26]. This phenomenon has implications for public health communication strategies, indicating that efforts to build trust in online vaccine information can have cascading effects on community engagement and knowledge dissemination, ultimately contributing to improved vaccination rates and public health outcomes [29].

The negative correlation (*r* = -0.026, *p* > 0.05) found between people’s reactions to vaccine availability in Malawi and their trust in online vaccine information is an interesting finding. On the surface, one might expect that higher trust in online vaccine information would correspond to more positive reactions toward vaccine availability. However, this result is consistent with studies that highlight the complexity of public attitudes toward vaccination, often influenced by contextual and socio-cultural factors [30, 31]. This unexpected finding prompts an exploration into the factors influencing public sentiment in the context of vaccine availability. Factors such as misinformation, fear of side effects, and cultural beliefs surrounding vaccination play a crucial role in shaping vaccine perceptions among Malawians, contributing to vaccine hesitancy and lower uptake rates compared to the set targets [5, 6].

Additionally, a recent study on the impact of social media news on COVID-19 vaccine hesitancy and vaccination behavior suggests that individuals are more sensitive to vaccine risk news than safety news on social media, indicating a relationship between the type of information and its impact on perception [17]. This resonates with the findings of this study, highlighting the complex nature of public sentiment, shaped by the interaction of trust, engagement, and the specific content of vaccine-related information on social media.

Moreover, although the negative correlation between COVID-19 vaccination and participation in COVID-19 discussions on social media (*r* = -0.087, *p* > 0.05) was not statistically significant, it indicates a possible a potential trend worth exploring further. This finding suggests that people who have received the COVID-19 vaccine may show slightly lower levels of participation in COVID-19 discussions on social media. This observation raises questions about the factors influencing online engagement among vaccinated individuals within this population group. One of the possible factors contributing to this trend could be that vaccinated individuals may feel a reduced sense of urgency or concern about COVID-19 compared to unvaccinated individuals, leading to less active participation in discussions about the virus on social media.

This study underscores the interaction between trust in online vaccine information, social media engagement, and public perception regarding COVID-19 vaccination. The positive correlation identified between trust and active participation in social media discussions highlights the role of reliable online sources in shaping public discourse. The negative correlation between trust and individuals’ reactions to vaccine availability prompts a deeper exploration into the factors influencing public perception. By acknowledging and addressing these factors, policymakers and healthcare providers can enhance vaccine acceptance and uptake rates.

### Limitations and future directions

While the study provides valuable insights, certain limitations warrant acknowledgment. The reliance on self-reported data and the cross-sectional design inherent in the methodology limit the extent to which causal inferences can be drawn [32]. Additionally, the exclusive focus on students from MUBAS may not fully capture the broader spectrum of the population. To address these constraints, future research could employ longitudinal designs and incorporate diverse demographic groups for a more comprehensive understanding of the dynamics shaping COVID-19 vaccine perceptions.

## Conclusions

The findings of this study underscore the importance of promoting trust in online vaccine information and leveraging digital platforms, particularly social media, to enhance health literacy and influence public health behaviors. Addressing vaccine hesitancy requires tailored communication strategies that are responsive to widespread concerns. By actively promoting trust in the veracity of online vaccine information and recognizing the influence of contextual and socio-cultural factors on public sentiment, public health campaigns can effectively utilize social media platforms to promote positive attitudes and perceptions regarding COVID-19 vaccination.

## Data Availability

The data obtained from the project is accessible and can be provided by the first author upon reasonable request.

## Abbreviations

COVID: 19–Coronavirus Disease 2019
MUBAS: Malawi University of Business and Applied Sciences
