# Correction: social media influence on COVID-19 vaccine perceptions among University students: a Malawi case study

**DOI:** 10.1186/s12889-024-18884-1

**Published:** 2024-05-27

**Authors:** Mervis Folotiya, Chimwemwe Ngoma

**Affiliations:** 1 Malawi University of Business and Applied Sciences, Blantyre, Malawi; 2 Department of Research and Innovation, ThinkSmart Consulting, Lilongwe, Malawi

BMC Public Health (2024) 24:1312.

10.1186/s12889-024-18764-8.

In the original publication of this article Tables 1 and 2 were incorrectly swapped. The original publication has been updated to rectify this error.

